# Comparative evaluation of nano and bulk tin dioxide cytotoxicity on dermal fibroblasts by real-time impedance-based and conventional methods

**DOI:** 10.3906/biy-1802-97

**Published:** 2018-10-25

**Authors:** Şükran ŞEKER

**Affiliations:** 1 Ankara University, Stem Cell Institute , Ankara , Turkey; 2 Tissue Engineering, Biomaterials and Nanobiotechnology Laboratory, Faculty of Science, Ankara University , Ankara , Turkey

**Keywords:** Tin oxide nanoparticles, dermal fibroblasts, cytotoxicity, real-time impedance measurement, cell index, nanoparticle aggregation, lactate dehydrogenase, MTT

## Abstract

In this study, the possible cellular effects of tin dioxide (SnO_2_) nanoparticles, together with its bulk form, on mouse dermal fibroblasts (DFs) were revealed using in vitro assays. Particle characterizations were carried out with AFM, Braun-Emmet-Teller, and DLS analyses. The cells were treated with nano and bulk SnO_2_ at concentrations of 0.1, 1, 10, 50, and 100 μg/mL for 6, 24, and 48 h. At the end of the exposure periods, the morphology, viability, particle uptake, and membrane leakage statuses of the cells were evaluated. Furthermore, real-time monitoring of cell responses was performed by using an impedance-based label-free system. Findings showed that at concentrations of 0.1-10 μg/mL, cells had similar doubling time to that of control cells (20.4 ± 2.6 h), while the doubling time of cells exposed to 100 μg/mL of nano and bulk SnO_2_ increased slightly (P ˃ 0.05) to 25.1 ± 3.9 h and 26.2 ± 5.9 h, respectively. The results indicated that DFs exhibited a similar toxicity response to nano and bulk SnO_2_; thus, 50 and 100 μg/mL of nano and bulk SnO_2_ had mild toxic effects on DFs. In conclusion, this study provides information and insight necessary for the safe use of SnO_2_ in medical and consumer products.

## 1. Introduction

Metal oxide nanoparticles (NPs) are increasingly taking place within various application fields of life sciences, materials science and engineering, and chemistry. The ascending use of NPs eventually leads to increased dermal exposure, constituting a potential risk to people subjected to them. Therefore, evaluation of potential toxic hazardous metal oxide NPs is crucial for human health. Tin oxide (SnO_2_) is an essential metal oxide semiconductor with a stable n-type wide band gap (3.6 eV). SnO_2_ has been widely used in numerous fields, including gas leakage detection, solar cells, catalysis, environmental monitoring, and chemical sensors (Roopan et al., 2015) . Besides, SnO_2_ NPs can be used for the cleansing of water contaminated with dye in the wastewaters of textile factories since they can play a role as photocatalysts for the removal of dye pigments. Despite their widespread use in many fields, in vitro cellular studies evaluating the safety/toxicity issues of SnO_2_ NPs for the mammalian system are very limited (Roopan et al., 2015; Tammina et al., 2017) . Thus, there are a few studies that have focused on the toxicity of nanosized SnO_2_ in bacterial systems ( Hu et al., 2009 ; ChávezCalderón et al., 2016 ) and marine organisms (Falugi et al., 2012; Gambardella et al., 2014) . To my knowledge, the potential toxic effects of SnO_2_ NPs on dermal fibroblasts have not been previously studied.

The most widely used colorimetric assays for the in vitro toxicity assessment of NPs, such as the ones based on reactive oxygen species, lactate dehydrogenase (LDH), and 3-(4,5-dimethylthiazol-2-yl)-2,5-diphenyl tetrazolium bromide (MTT), have the possibility to interfere with NPs, which have high absorption or scattering properties. Furthermore, because of their large surface area and high surface energy, NPs can adsorb the test reagents used in the detection or labelling steps, which may result in false negative or positive outcomes (Kroll et al., 2012) . In addition, these techniques cannot monitor the cell responses dynamically following exposure to NPs. On the contrary, impedance-based high-throughput instruments for in vitro analysis are reliable and efficient label-free devices for determination of cell responses in real time ( Dönmez Güngüneş et al., 2017 ). Recently, many studies have revealed the responses of cells to NPs using the impedance-based system. For example, the cytotoxic responses of bronchial epithelial cells, Chinese hamster ovary cells, and human embryonic kidney cells to citratestabilized gold NPs were successfully assessed by the impedance-based technique (Vetten et al., 2013; Pisani et al., 2017) . Carbon nanotubes with different diameters and surface functionalization were tested for their potential toxic effects to five different cell lines: DMBM-2 mouse macrophages, murine L929 and V79 cells, Chinese hamster lung fibroblasts, endothelial EAhy926 cells, and human MRC-5 fibroblasts (Meindl et al., 2013) . The toxic effects of eleven inorganic nanomaterials to human bronchial epithelial cells were monitored in real time ( OteroGonzález et al., 2012 ). Moreover, the viability of A549 cells exposed to ZnO NPs or Al-ZnO NPs was monitored by the impedance-based system (Pan et al., 2014) . Another in vitro study has revealed the cytotoxicity of different cell lines (A549, B16, and C6) treated with ciprofloxacininhibited topoisomerase II in eukaryotes (Kloskowski et al., 2012) . The effects of paclitaxel on MDA-MB-231 breast cancer cells and A549 lung cancer cells were assessed by both impedance-based and classical toxicity assays (Limame et al., 2012) .

In this study, the potential toxic responses of dermal fibroblasts against nano and bulk SnO_2_ were investigated by using a real-time cell impedance system in addition to conventional methods, i.e. the mitochondrial activity (MTT assay) and membrane leakage (LDH assay). To characterize the physicochemical properties of nano and bulk SnO_2_, the zeta potential, particle size distribution, and mean surface area were determined using dynamic light scattering (DLS) and Braun–Emmet–Teller (BET) analyses. The subcellular localization of nano and bulk SnO_2_ was evaluated by transmission electron microscopy (TEM).

## 2. Materials and methods

### 2.1. Chemicals

All chemicals and solvents were of analytical grade. Nano and bulk tin oxide and the assay kits (MTT and LDH) were purchased from Sigma-Aldrich (St. Louis, MO, USA). Purchased nano and bulk tin oxide were characterized in detail before use.

### 2.2. Atomic force microscopy (AFM)

Nano and bulk SnO_2_ were dissolved in dichloromethane (Sigma) at 25 °C and disaggregated using an ultrasonic bath (Fisher FB15060, Schwerte, Germany). Thin coatings were casted on polished gold surfaces using a spin coater (Primus SB15, Singen, Germany) (Şeker et al., 2010). The coatings were scanned with 40 N/m force constant using an atomic force microscope (Nanomagnetics Inst., Ankara, Turkey) and imaged at a scan area of 3 × 3 µm (Şeker et al., 2016). Two-dimensional, three-dimensional, and crosssectional profiles of coated surfaces were obtained.

### 2.3. Braun–Emmet–Teller analysis

Mean surface areas of nano and bulk SnO_2_ were determined by BET analyses. Nitrogen adsorption/desorption measurements were performed using a volumetric gas adsorption device (Quantachrome Instruments, Boynton Beach, FL, USA).

### 2.4. Preparation of nano and bulk SnO_2_ dispersions

Before preparation of the nano and bulk SnO_2_ dispersions, the particles were sterilized by UV irradiation (254 nm) for 30 minutes. Stock dispersions of sterile particles were prepared in cell medium and sonicated using ultrasonic bath for 30 min. Particle suspensions at different concentrations (0.1, 1, 10, 50, and 100 μg/mL) were prepared by diluting the dispersed stock solutions with cell culture medium.

### 2.5. DLS analysis

To determine the surface charge and average particle size of the nano and bulk SnO_2_ with a polydispersity index, particle suspensions (at concentration of 100 μg/mL) were dispersed in the cell medium using the ultrasonic bath before DLS experiments. The measurements were sequentially performed in replicates of thirteen for each particle using a NanoZetasizer-ZS model instrument (Malvern, Worcestershire, UK) at 25 °C.

### 2.6. Culture of dermal fibroblasts

Cells purchased from ATCC were thawed and then cultured in DMEM containing 10% fetal bovine serum (FBS), 2 mM L-glutamine, 100 U/mL penicillin, 100 μg/ mL streptomycin, and 1% nonessential amino acids (all from Lonza, Basel, Switzerland) in a standard humidified cell culture incubator with 5% CO2 at 37 °C. After 2 days of culture, nonadherent cells were removed by washing gently with sterile PBS. The cell medium was replaced twice a week.

### 2.7. Nano and bulk tin oxide exposure studies

For the tin oxide exposure experiments, DFs were incubated with different concentrations (0.1, 1, 10, 50, and 100 μg/mL) of nano and bulk SnO_2_ for 6, 24, and 48 h. Briefly, when the cells reached ~70% confluence, they were harvested by using 0.25% trypsin-EDTA, suspended in cell culture medium, counted by a hemocytometer, and then seeded at a density of 1 × 10^4^ cells/well into 96-well plates. The seeded cells were cultured for 24 h in the incubator at 37 °C and 5% CO2 to allow DFs to attach to the wells. The cell culture medium was then removed and the nano and bulk SnO_2_ suspensions were added into each well. Cells were cultured in tin oxide containing medium for 6, 24, and 48 h. For control experiments, the cells were cultured at the same seeding density without any particles.

### 2.8. Cell morphology

After 48 h of particle exposure, the morphological changes on dermal fibroblasts treated with different concentrations of nano and bulk SnO_2_ were monitored under an inverted microscope (Zeiss, Jena, Germany). DFs cultured in standard medium without any particles served as control.

### 2.9. Subcellular localization of particles

The cellular uptake of nano and bulk SnO_2_ was demonstrated by TEM. Briefly, the cells were cultured in 6-well plates at a density of 0.3 × 10^6^ cells/well. When DFs reached a confluence of ~70%, nano sized and bulk SnO_2_ (100 μg/mL) were added into each well and incubated for 48 h. Unexposed DFs were used as the control group. TEM samples were prepared according to the method reported elsewhere (Elcin et al., 2003). Samples were sectioned using an ultramicrotome (Leica, Ultracut UCT, Vienna, Austria), then stained with 1% toluidine blue, and then poststained with 4% uranyl acetate in methanol and Reynolds lead citrate solution. Stained sections were observed under a TEM instrument (JEOL 100 CX, Tokyo, Japan).

### 2.10. MTT assay

MTT assay was used to evaluate the viability of cells exposed to nano and bulk SnO_2_ in terms of mitochondrial activity. Cells cultured in a medium without any particles were used as a control group. After exposure to particles, the cells were gently rinsed with PBS three times to remove the residuals and then incubated with 20 μL of 5 mg/mL MTT solution and 180 μL of DMEM at 37 °C and 5% CO2 for 4 h. At the end of incubation, the medium was removed carefully; resultant formazan crystals were dissolved in 200 μL of MTT solvent. Then, the 96-well plates were centrifuged at 250 × g for 5 min. Supernatants were transferred to new 96-well plates. Absorbances were measured at a wavelength of 570 nm using a microplate reader (Molecular Devices, Sunnyvale, CA, USA). Relative cell viability (%) was calculated as: [A]_sample_/[A]_control_ × 100, where [A]_sample_ is the absorbance of the test sample, [A]_control_ is the absorbance of the control sample, [A]_sample_ is the absorbance of cells exposed to particles, and [A]_control_ is the absorbance of the control cells.

### 2.11. Membrane leakage assay

Lactate dehydrogenase released from the cells was determined by LDH Toxicology Assay kit (Sigma). As positive control, cells were incubated in 10 μL of lysis solution for 45 min before centrifugation. Then, 96-well plates were centrifuged at room temperature for 5 minutes at 250 × g. Then, 50 μL of supernatant and 100 μL of LDH assay solution were mixed in each well and incubated for 30 min. Fifteen μL of 1 N HCl were added to each well to stop the reaction. Absorbance values at 490 and 690 nm were measured using a microplate reader. Thereafter, the absorbance at 490 nm was subtracted from the absorbance of the same well measured at 690 nm. LDH leakage (% of positive control) was expressed as the percentage of ([A]_sample_ – [A]negative)/([A]_positive_ – [A]_negative_) × 100, where [A]_sample_ is the absorbance of cells exposed to particles, [A]_positive_ is the absorbance of the positive control cells, and [A]_negative_ is the absorbance of the cell medium.

### 2.12. Real-time cell impedance measurements

Impedance-based cell proliferation and cytotoxicity assays were performed using the xCELLigence Real-Time Cell Analyzer instrument (RTCA SP, Roche Applied Science, Penzberg, Germany).

#### 2.12.1. Cell proliferation assay

A preliminary cell proliferation assay was performed at different cell densities to determine the optimum cell density and culture duration to be used for the principal cytotoxicity assay. This step is necessary, since an optimal cell density is needed to ensure the reliability of the subsequent data. Briefly, for background measurement, cell culture medium (100 μL) was added into each well of E-plates (Roche Diagnostics, Basel, Switzerland). Then, cell suspensions (100 μL) were added into each well to obtain final concentrations of 100,000, 50,000, 25,000, 12,500, 6250, 3125, 1563, and 781 cells/well. The cell index (CI) values were continuously recorded at 15 min intervals for 25 h duration.

#### 2.12.2. Cytotoxicity assay

Nano and bulk SnO_2_ cytotoxicity on dermal fibroblasts was determined using the xCELLigence system in real time. Briefly, after background measurements with the culture medium, the DFs were added into the wells of each electrode-plate at a density of 6.2 × 10^3^ cells/well (optimized cell density). Then the DFs were cultured in a carbon dioxide incubator for 15 h (optimized duration) to allow the cells to attach to the E-plates. After 15 h of culture, nano and bulk SnO_2_ suspensions were added into each well (final concentrations of 0.1, 1, 10, 50, and 100 μg/mL). Control cells were exposed to the culture medium without any particles. Cell index measurements were performed at 15 min intervals for 72 h. The effect of particles on cell index measurements was investigated with nano and bulk SnO_2_ suspensions (without cells) with the highest concentrations used. Doubling times of the control and SnO_2_-exposed cells were calculated using the xCELLigence software (ACEA Biosciences, San Diego, CA, USA).

### 2.13. Statistical analysis

Results are expressed as the mean ± standard deviation. MTT and LDH experiments were performed in triplicates. Real-time cytotoxicity experiments were performed in quadruplicates. Comparisons between groups were analyzed by one-way or two-way ANOVA with Bonferroni posttest using the GraphPad Prism software (*P < 0.05, **P < 0.01, ***P < 0.001).

## 3. Results

### 3.1. AFM

**Figure 1:**
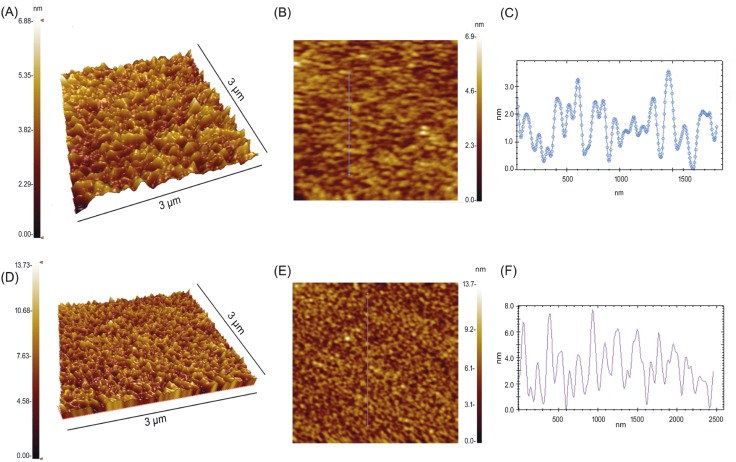
Three-dimensional and two-dimensional AFM images and cross-section profiles of the nano (A, B, C) and bulk SnO_2_ (D, E, F).

AFM technique was used to evaluate the topography of surfaces coated with nano and bulk SnO_2_. As shown in Figure 1, nano and bulk SnO_2_ films showed a homogenous rough morphology. Nano SnO_2_ film has a root-mean square (rms) roughness of 0.8 nm, whereas bulk SnO_2_ film has an rms roughness of 1.7 nm. Cross-section profiles of nano (Figure 1C) and bulk (Figure 1F) SnO_2_ determined from 3 × 3 µm scan areas showed that height values of the nano coating were in the range of ~1–3 nm, while the height values for the bulk coatings were ~2–7 nm.

### 3.2. BET results

The volumes of nitrogen gas adsorbed onto the nano and bulk SnO_2_ surfaces were determined by BET analyses. Specific surface areas of the particles are shown in Table. BET results indicated that nano SnO_2_ had higher surface area (55.448 m2/g) for the nitrogen to be adsorbed, compared to that of bulk SnO_2_ (1.445 m2/g). It can be seen from the results that due to the nano structure, nano SnO_2_ showed a higher BET surface area compared to the bulk form.

**Table:** Characterization of the nano and bulk SnO_2_.

	Nano SnO_2_	Bulk SnO_2_
BET results (m2/g)	55.448	1.445
Average diameter in DLS (nm)	365.2 ± 16.2	1050 ± 10.6
Zeta potential (mV)	17.1 ± 3.82	–21.4 ± 5.35
Polydispersity index	0.327 ± 0.08	0.323 ± 0.06

### 3.3. Particle size distribution

**Figure 2:**
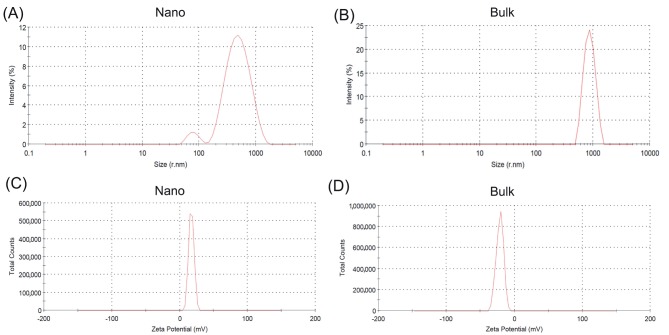
Size distribution (A, B) and surface charge (C, D) of the nano and bulk SnO_2_

The hydrodynamic diameter, polydispersity index, and zeta potential of the nano and bulk SnO_2_ in the cell culture medium were determined by the dynamic laser light-scattering particle size analyzer. Measurement results are presented in Figure 2 and Table. Nano and bulk SnO_2_ showed distinctive particle size distribution and average particle size. The average hydrodynamic diameter of the nano and bulk SnO_2_ retrieved from DLS analysis were 365.2 ± 16.2 nm and 1050 ± 10.6 nm, respectively. Figure 2A indicated that nano SnO_2_ showed a wider particle size distribution. The zeta potential of particles inside the cell culture medium was found to be 17.1 ± 3.82 for the nano SnO_2_, and −21.4 ± 5.35 for the bulk SnO_2_. According to the zeta potential results, the absolute value of the zeta potential for bulk SnO_2_ was higher than that of the nano SnO_2_. Due to the fact that the electrostatic repulsive force among the particles was higher, bulk SnO_2_ was colloidally more stable in solution.

### 3.4. Cell morphology

**Figure 3:**
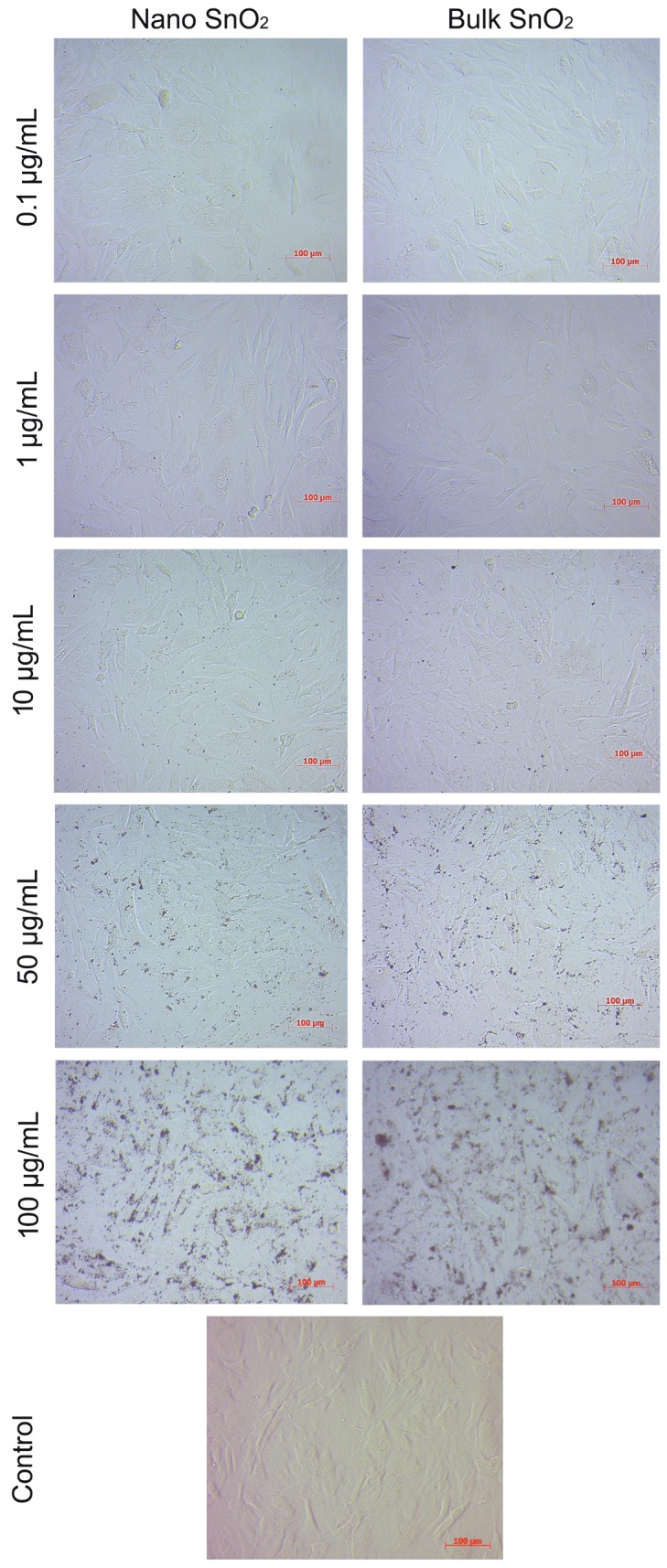
Inverted phase-contrast micrographs of dermal fibroblast cultures following exposure to different concentrations of nano and bulk SnO_2_ and control cells. Scale bars: 100 μm.

The morphology of DFs exposed to SnO_2_ NPs and SnO_2_ bulk particles for 48 h, and that of the control cultures, are shown in Figure 3. In the nano- and bulk-exposed groups, there was no dramatic morphological difference at the tested concentrations. On the side, both nano and bulk SnO_2_ particles were observed to attach to the cell surface, especially at high concentrations.

### 3.5. Subcellular localization of nano and bulk SnO_2_

**Figure 4:**
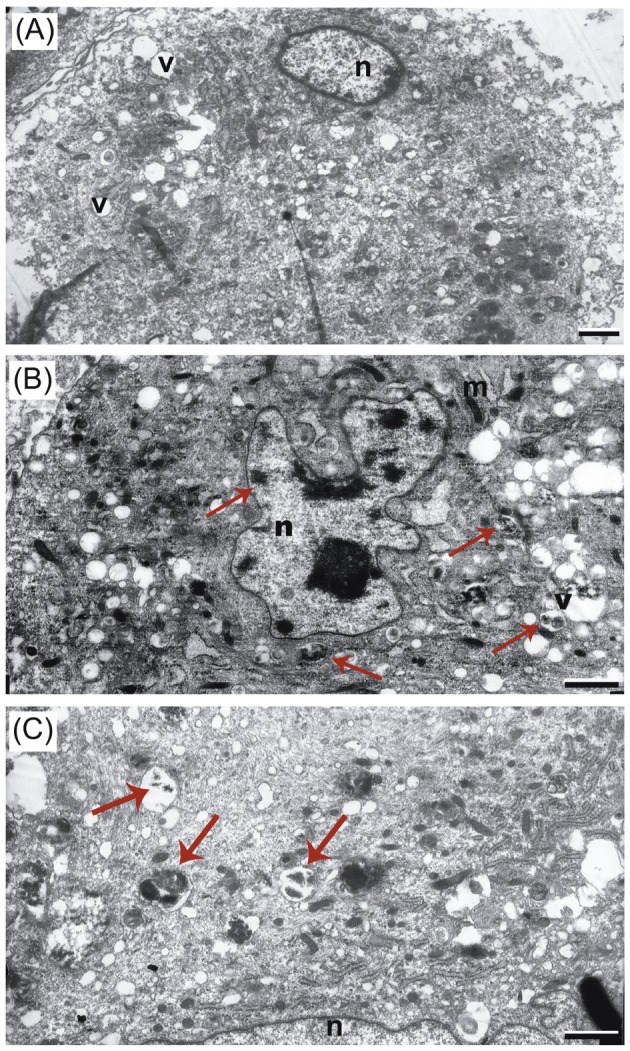
TEM micrographs of dermal fibroblasts: control cell (A), nano SnO_2_-treated DF (B), and bulk SnO_2_-treated DF (C). Arrows point to particle agglomerates. Abbreviations: v, vesicle; n, nucleus; m, mitochondria. Scale bars = 1 μm.

Figure 4 shows the TEM images of unexposed (A), nano SnO_2_ exposed (B), and bulk SnO_2_ exposed (C) DFs. According to TEM analyses, both nano and bulk SnO_2_ were mostly located in the cytoplasmic vacuoles and the nucleus, while particles were not observed in the vesicles of the control cells as expected. In addition, nano and bulk SnO_2_ tended to aggregate in the cytoplasm and in the nucleus after 48 h of incubation with the DF cells. Figure 4B demonstrates that the shape of DF cell nucleus was significantly distorted.

### 3.6. Cell viability results

**Figure 5:**
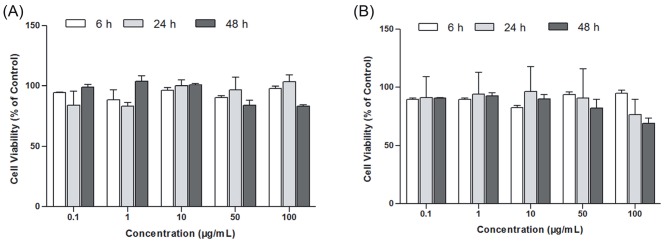
Effect of nano (A) and bulk SnO_2_ (B) on the viability of dermal fibroblasts exposed to 0.1–100 μg/mL of SnO_2_ for 6, 24, and 48 h.

Mitochondrial activity of the DFs was determined by MTT assay following 6, 24, and 48 h of exposure to nano and bulk SnO_2_. Cell viability results are presented in Figure 5. It is clear from the figure that the nano and bulk SnO_2_ did not have a significant toxic effect on DFs after 6 h of exposure. Also, nano and bulk SnO_2_ showed no significant effect on the viability of cells at concentrations below 50 μg/mL. The viability of DFs exposed for 48 h to either nano or bulk SnO_2_ at the concentration range of 50–100 μg/mL showed a decrease compared to that of the control group.

### 3.7. LDH measurements

**Figure 6:**
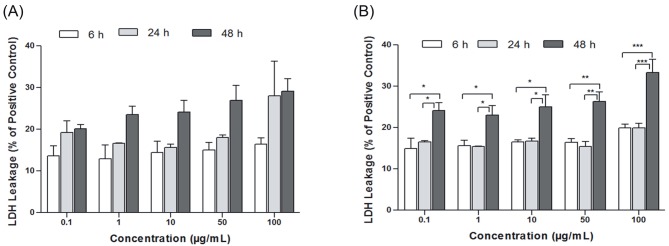
Change in LDH activity of dermal fibroblasts exposed to 0.1–100 μg/mL of nano (A) and bulk SnO_2_ (B) for 6, 24, and 48 h.

LDH release, an indicator of cell membrane damage, was investigated on exposed and nonexposed cell cultures. After 6 h of incubation with nano SnO_2_, the level of LDH release from the cultured cells was nearly the same at all concentrations (Figure 6A). However, the level of LDH release after 48 h of nano SnO_2_ exposure was significantly higher than that of cultures after 6 h of exposure. It was found that nano SnO_2_ led to concentration and timedependent increase in LDH level (Figure 6A). Figure 6B shows that after 6 h of incubation with bulk SnO_2_, the level of extracellular LDH was similar to that of the 24-h exposure. At longer incubation time, the amount of extracellular LDH increased significantly in the presence of bulk SnO_2_ (Figure 6B).

### 3.8. Cell index measurements

**Figure 7:**
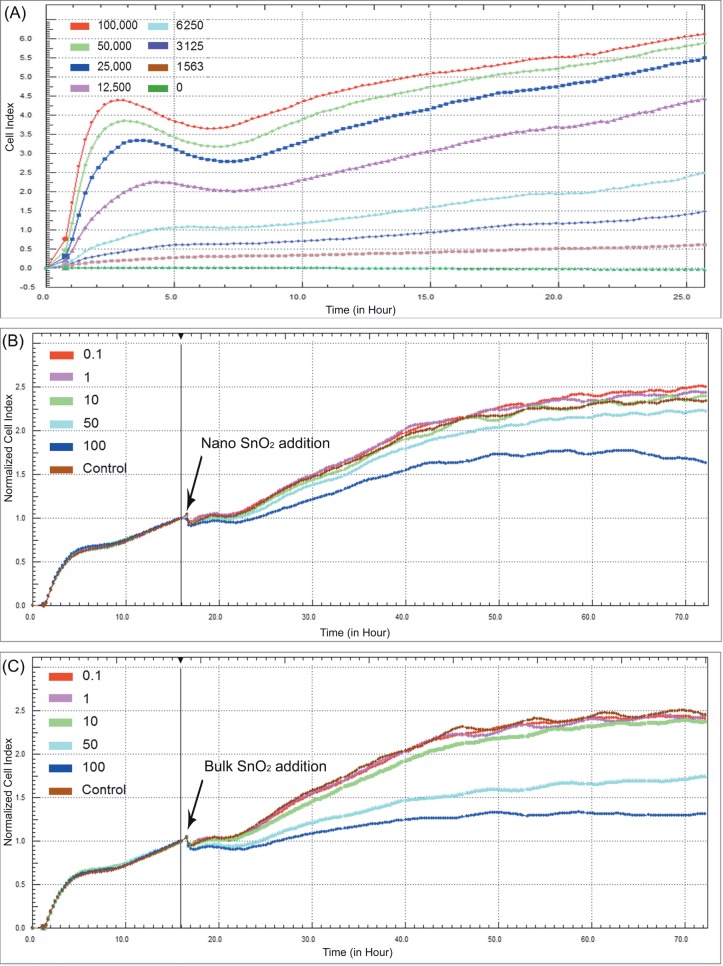
Real-time monitoring of the adhesion and proliferation of dermal fibroblasts at different seeding densities (A). Real-time monitoring of cell proliferation following exposure to nano (B) and bulk (C) SnO_2_ using the impedance system.

Electrical impedance measurements of DFs at different concentrations are shown in Figure 7A. The results of DF proliferation experiments indicate a concentration-dependent exponential increase in CI values. Results also revealed that the suitable cell number and time needed to reach the mid-log phase were 1.25 × 10^4^ cells/well and 15 h, respectively.

Normalized cell index (NCI) values of nano and bulk SnO_2_-exposed cells are shown in Figures 7B and 7C, respectively. At concentrations below 50 µg/mL, both nano and bulk SnO_2_-exposed cells showed NCI patterns quite similar to those of the control cells. By contrast, the rise of the NCI pattern slowed down for DFs treated with bulk SnO_2_ at concentrations of 50–100 μg/mL, and a decline in NCI was observed ∼7 h after the addition of the particles. After 48 h of exposure to SnO_2_ NPs, NCI had decreased by ~10% in 50 μg/mL, ~30% in 100 μg/mL, while exposure to bulk SnO_2_ resulted in a decrease by ~30% in 50 μg/mL, and ~45% in 100 μg/mL. It is clearly seen that bulk SnO_2_ at 50 and 100 μg/mL caused a higher level of decrease in the cell index compared to that of nano SnO_2_.

**Figure 8:**
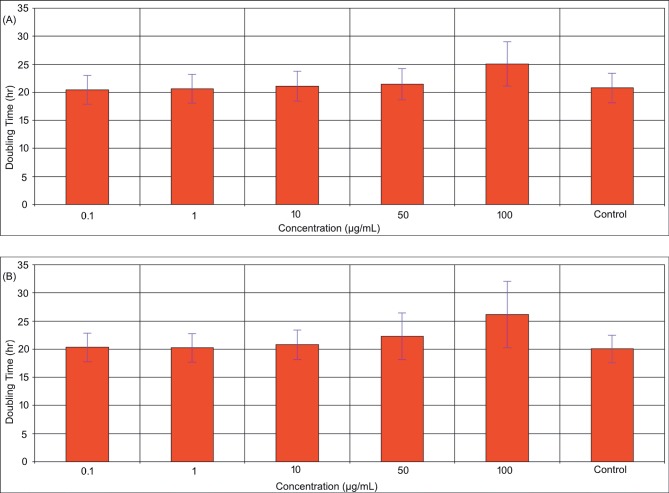
Average doubling time of the dermal fibroblasts exposed to different concentrations of nano (A) and bulk (B) SnO_2_.

Doubling times (in hours) of the control and SnO_2_exposed cells were determined using xCELLigence RTCA software. While the calculated doubling time for the cells exposed to 100 µg/mL nano SnO_2_ was 25.1 ± 3.9 h, it was 26.2 ± 5.9 h for the bulk SnO_2_ exposure (Figure 8). The results indicated that at concentrations up to 50 μg/mL of nano and bulk SnO_2_, the cells had similar doubling times with the control cells (20.4 ± 2.6 h). A slight increase in the doubling time at 100 µg/mL was observed after incubation with nano and bulk SnO_2_. According to applied one-way ANOVA, no significant difference in doubling times was obtained between the groups (P ˃ 0.05).

## 4. Discussion

Metal oxide NPs have been widely used in the fields of cosmetics, pharmaceuticals, and in other industrial applications due to their exceptional physicochemical properties. A crucial role of metal oxide NPs in the biomedical field has been revealed in relation to their unique structure, high surface area, biocompatibility, interesting redox and catalytic properties, and good mechanical stability (Andreescu et al., 2012) . With the growing number of their applications, detailed toxicological evaluation of metal oxide NPs has become mandatory to assess their risks to human health and environmental safety ( Oberdörster et al., 2007 ).

NPs may be able to directly surpass the skin, which is the largest organ of the human body. Important routes for NP entry into the skin are the use of cosmetics, accidental exposure, and other local applications. Therefore, the interaction of NPs with the skin is an important issue (Mortensen et al., 2010) . In the present study, therefore, dermal fibroblasts were used in the in vitro toxicology experiments.

It is well known that NPs tend to aggregate and accumulate more rapidly in suspensions leading to the formation of larger particles; thus, this property may effect the level of toxicity (Z hu et al., 2009 ). In this study, the hydrodynamic size of the nano and bulk SnO_2_ in cell culture medium was determined. The resulting particle size distribution measurements retrieved from the DLS analyses indicated that nano and bulk SnO_2_ formed distinctive aggregates and sizes in cell culture medium. The size of bulk SnO_2_ in solution was greater than the size of SnO_2_ NPs.

To understand the interaction of NPs with biological systems, the physicochemical properties of NPs, such as polydispersity index, surface area, and surface charge should be comprehensively investigated (Paik et al., 2015) . The polydispersity index of nano and bulk SnO_2_ suspensions (<1) indicated a highly unstable particle suspension (Şeker et al., 2014). Specific surface areas of nano and bulk SnO_2_ were determined by gas adsorption using the BET method. According to BET results, nano SnO_2_ had a larger surface area per mass than its bulk form, as expected. A higher surface area may lead to higher reactivity with nearby particles, resulting in possibly toxic effects (Shin et al., 2015) . However, in suspensions, since NPs tend to form aggregates, it is possible that the bulk form may show a comparable level of toxicity to the nano form (Xiong et al., 2011) . This statement is in agreement with the present results. This may be part of the explanation for similar toxic effects of nano and bulk SnO_2_ observed in the study.

The effect of nano and bulk SnO_2_ exposure on the viability of cells was evaluated by MTT assay. The results showed that exposure to nano and bulk SnO_2_ caused similar cytotoxic responses of the DFs (Figure 5). Exposure of cells to nano and bulk SnO_2_ up to a concentration of 100 μg/mL did not induce any significant effect on cell viability at 6 and 24 h, despite having membrane damage, which was detected by the LDH assay (Figure 6). This result can be explained by the fact that SnO_2_ exposure has caused membrane leakage, but the cells have managed to retain their viability by the repair mechanisms of the plasma membrane (Draeger et al., 2011). However, when exposure time was increased to 48 h, nano and bulk SnO_2_ at 100 μg/ mL led to a higher decrease in cell viability together with an increase in LDH leakage.

TEM is a major method for determination of the possible fates of NPs after entry into the cell, which could indicate the localization of NPs (Schrand et al., 2010) . Subcellular localizations of nano and bulk SnO_2_ were shown using TEM analysis. It was observed that nano and bulk SnO_2_ were taken up by the dermal fibroblasts after 48 h of incubation. As seen in Figure 4, both nano or bulk particle aggregates were mostly internalized in the cytoplasmic vesicles and the nucleus. No particles were observed in the cytosol or in other cell organelles of the control cells, as expected.

Recently, impedance-based methods, which allow label-free and real-time detection, have gained importance for determination of cell responses to substances due to advantages over traditional cytotoxicity assays (Pisani et al., 2015) . In this study, in order to reveal the cytotoxic effects of the nano and bulk SnO_2_, CI measurements of the DFs seeded on electrodes were carried out for 72 h. Results obtained from the xCELLigence system corresponded well with those of the MTT and LDH assays. The impedance data showed that both types of SnO_2_ particles at concentrations of 50–100 μg/mL exhibited a decrease in NCI. The NCI values of DFs treated with SnO_2_ NPs did not show a significant change at concentrations up to 50 μg/mL, which was substantially similar to that of the control cells. DF cell growth inhibition was observed at 50 and 100 μg/mL of exposure for both particle types. However, a decrease in the proliferation rate as measured by impedance-based system was higher in 50 and 100 µg/mL bulk SnO -exposed DFs compared to nano SnO_2_2 exposed DFs (Figure 7).

Results in Figure 8 indicated that cells exposed to nano and bulk SnO_2_ had similar doubling times under the experimental conditions. No significant difference in doubling times was observed in either nano or bulk SnO_2_ groups. Cells exposed to 50 and 100 µg/mL nano and bulk SnO_2_ had longer doubling times than the control cells (P ˃ 0.05). Exposure to either type of SnO_2_ particles led to a slight increase in the doubling time of DFs after 90 h of exposure at the highest dose (100 µg/mL).

To my knowledge, to date there is no study evaluating the cytotoxic effects of nano and bulk SnO_2_ on dermal fibroblasts. Roopan et al. (2015) have searched for the toxic effect of SnO_2_ NPs synthesized by using the extract of Annona squamosa that was evaluated on HepG2 cells and the results showed moderate toxicity of SnO_2_ NPs to this cell type. In another study, the cytotoxic effect of SnO_2_ NPs synthesized by using Piper nigrum on HCT116 and A549 carcinoma cells was investigated and the results indicated that SnO_2_ NPs had a higher toxic effect on A549 than the HCT116 cancer cell lines (Tammina et al., 2017) . In the present study, comparable toxic effects of nano and bulk SnO_2_ on dermal fibroblasts have been reported at the tested concentrations. In fact, NPs are theoretically expected to be more toxic than their bulk forms due to their higher reactivity, higher surface area, and their capacity to penetrate easily into the cell membrane and accumulate inside the cells. Nevertheless, based on the obtained results, SnO_2_ in the form of NPs had a similar toxic effect compared to its bulk form. Consistent with previous studies (Huk et al., 2014) and as shown in the obtained results, it cannot be easily generalized that NPs are more toxic than their bulk form. As these results indicate, not only size plays a major role in the fate and toxicity of NPs, but also chemical composition, aggregation, and surface functionalization are essential factors that could effect the toxicity of NPs (Keck and Müller, 2013) .

This study evaluated the potential toxic effects of SnO_2_ NPs together with its bulk form on dermal fibroblasts by using an impedance-based system. Nano and bulk SnO_2_ had no significant toxic effects on fibroblasts at concentrations below 50 μg/mL, while it demonstrated a low level of toxicity at 50 and 100 μg/mL concentrations. The present study has attempted to address the significant lack of toxicology data for SnO_2_ NPs on dermal fibroblasts. This study could be helpful for evaluating and making a comparison between nano and bulk SnO_2_ in vitro toxicity on dermal fibroblasts.

## Acknowledgments

I thank Prof. Y. Murat Elçin and Prof. A. Eser Elçin very warmly for their advice and support.
